# Beclin1-mediated vascular autophagy negatively regulates angiogenesis and secondary neural damage in the thalamus following cerebral cortical infarction

**DOI:** 10.1016/j.ibneur.2025.07.008

**Published:** 2025-07-19

**Authors:** Mengzhi Liu, Yuqian Chen, Xinyan Fan, Jinmin Gu, Shihui Xing

**Affiliations:** a Department of Neurology and Stroke Center, The First Affiliated Hospital of Sun Yat-sen University, Guangzhou, Guangdong, China; b Guangdong Provincial Key Laboratory of Diagnosis and Treatment of Major Neurological Diseases, National Key Clinical Department and Key Discipline of Neurology, Guangzhou, Guangdong, China

**Keywords:** Angiogenesis, Autophagy, Beclin1, Cerebral infarction, Thalamus

## Abstract

Focal cerebral infarction induces angiogenesis in the thalamus, which influences cognitive recovery. However, the mechanisms of angiogenesis in the thalamus remain unclear. This study was designed to investigate the potential role of Beclin1-mediated vascular autophagy in angiogenesis occurring in the thalamus after cerebral infarction. Cerebral infarction was induced by middle cerebral artery occlusion (MCAO). Cognitive function was evaluated using the Morris Water Maze. We assessed secondary neuronal damage, angiogenesis, Beclin1 expression and vascular autophagy in blood vessels of the ipsilateral thalamus. The functional effects of Beclin1 on vascular autophagy, angiogenesis and angiogenesis-related factors were determined using lentiviral-delivered siRNA. The results revealed significant angiogenesis in the ipsilateral thalamus at 7 days after MCAO, concurrent with elevated LC3-I to LC3-II conversion and increased Beclin1 expression in the ipsilateral thalamic vessels. Knockdown of Beclin1 markedly suppressed vascular autophagic activation and potentiated thalamic angiogenesis at the above time point. This enhanced angiogenesis correlated with significant reductions of neuronal loss and astrogliosis in the ipsilateral thalamus, alongside improved cognitive function. Furthermore, Beclin1 knockdown significantly increased the levels of angiopoietin-2 (ANG-2) and vascular endothelial growth factor (VEGF) in the ipsilateral thalamus after cerebral infarction. Collectively, these findings implicate that inhibition of Beclin1-mediated vascular autophagy enhances angiogenesis and mitigates secondary thalamic neuronal damage following cerebral infarction. This neuroprotective effect likely related to the restoration of ANG-2 and VEGF levels mediated by vascular autophagy in the thalamus.

## Introduction

In addition to primary situ insults, focal cerebral infarction can induce secondary neural damage in remote regions connected to the ischemic focus via fibers such as the thalamus (Ogawa et al., 1997, Zhang et al., 2012a). Clinical and experimental studies have confirmed that the extent of such secondary damage is correlated with the degree of neurological deficits after ischemic stroke (Baron et al., 2014, Kuchcinski et al., 2017). Comparatively, blockade of the secondary damage has been shown to promote neurological functional recovery in experimental ischemic stroke (Zhang et al., 2012b). Different from neural injury in the primary infarct regions, there is a significant temporal delay onset of remote secondary damage, thus providing a relatively wide therapeutic window.

Previous studies have shown that stroke triggered endogenous angiogenesis in addition to neural damage (Arai et al., 2009, Hatakeyama et al., 2020). Using MRI approach, a gradual increase in vascular density was seen in brain regions outside the primary infarct site (Yanev et al., 2017). As a potential repair mechanism, angiogenesis is suggested to facilitate the clearance of harmful factors and damaged tissues. Clinical studies have revealed a positive correlation between angiogenesis and neural plasticity, long-term survival time and functional recovery after stroke (Lake et al., 2017). The sustained increase of blood flow as resultant of angiogenesis would promote neural structural remodeling and functional recovery, thereby mitigating the secondary neurological damage at remote brain regions (Surugiu et al., 2018). Previously, we have demonstrated that activation of ephrinB2 signaling enhanced angiogenesis and attenuated the neural injury in the thalamus after ischemic stroke, indicating a protective effect of angiogenesis against the secondary thalamic damage (Xing et al., 2019).

Autophagy has been indicated in the regulation of angiogenesis under different pathophysiological situations. It has been shown that autophagy exerted a promotive effect on angiogenesis during embryonic vascular development, and pharmacological inhibition of autophagy resulted in embryo demise by disrupting the process of angiogenesis (Lu et al., 2016). This is supported by other study showing that knockdown of autophagy-related genes 3/5 promoted angiogenesis (Hughes et al., 2020). Recently, we have shown that blockade of Nogo-A/S1PR2 suppressed autophagic activation in blood vessels and promoted angiogenesis in the ipsilateral thalamus after cerebral infarction (Xiao et al., 2022). However, it remains unclear how autophagy regulates angiogenesis in the thalamus following distal cerebral infarction.

Beclin1 is a key autophagy regulator and serves as a scaffold for Class III phosphatidylinositol 3-kinase complexes to modulate lipid kinase activity through allosteric regulation (Tran et al., 2021). Beclin1-mediated autophagy is closely linked to angiogenesis. Upregulation of Beclin1 would lead to the activation of autophagy and inhibit angiogenesis in tumor tissues. Conversely, knockdown of Beclin1 significantly enhanced tumor angiogenesis (Rybstein et al., 2018). However, whether Beclin1-mediated autophagy was involved in angiogenesis of the thalamus is required to be further elucidated.

In this study, we aimed to investigate the potential roles of Beclin1-related autophagic activations in blood vessels on angiogenesis in the thalamus after cerebral infarction. Further, we explored the possible mechanisms of angiogenesis regulated by Beclin1-mediated autophagy.

## Materials and methods

### Animal models

Four-week-old male Sprague-Dawley rats, weighing 80–100 g, were purchased from Guangdong medical laboratory animal center (Guangdong, China). Stroke-prone renovascular hypertensive rats (RHRSP) model was established using a two-kidney two-clip approach as previously described (Zeng et al., 1998). At approximate 10 weeks post-surgery, animals exhibiting systolic blood pressure over 180 mmHg without any neurological symptom were selected for the next experiments. The experimental study was approved by the Institutional Animal Ethical Committee of Sun Yat-sen University.

A group of RHRSPs was randomly classified to receive middle cerebral artery occlusion (MCAO) or sham-operated surgery (9 animals per group). MCAO was introduced according to the protocol as previously described (Brint et al., 1988). In brief, under anesthesia with 3 % isoflurane, the right middle cerebral artery (MCA) was exposed by removal of the squamous portion of the temporal bone. Subsequently, the right MCA was occluded at the site distal to the origin of the striatal branches with a bipolar electrocoagulation device under an operating microscope. RHRSPs in the sham-operated group underwent the same procedure except for the occlusion of the MCA. Another group of RHRSPs was randomly treated with stereotactically guided delivery of *Becn1*-siRNA or Scramble-siRNA lentiviral vectors into the right thalamus 2 weeks prior to MCAO operation (9 animals per group). A total of 6 animals were excluded from the study due to intracranial hemorrhage or death during operations. The necessary sample size was estimated with an α-level of 0.05 and a desired power (1-β) of 0.80, based on our and other publications (Dirnagl, 2006, He et al., 2007, Ling et al., 2009, Zhang et al., 2011, Chen et al., 2017). The sample size of 9 animals in each group has a power of 93.3 % to detect the group difference.

### SiRNA preparation and intrathalamic injection

The siRNA sequences targeting rat Beclin1 (GenBank, Accession NM-053739) and a negative control sequence were constructed by Genechem (Shanghai) according to our previous study (Xing et al., 2012). The best-performing sequence of siRNA targeting *Becn1* was *GAGGAGCCATTTATTGAAA*, and a scrambled sequence of *TTCTCCGAACGTGTCACGT* was designed for negative control. SiRNA sequences were inserted into a pFU-GW-iRNA lentivirus vector, containing a CMV-driven EGFP reporter gene and a U6 promoter upstream of restriction sites (*Age*I and *Eco*RI) (Genechem). Thereafter, recombinant lentivirus was produced by co-transfecting 293 T cells according to the standard protocol. GFP expression in 293 T cells was measured to determine the virus titers. The final titers were approximately 3 × 10^9^ transducing units/milliliter. The delivery of lentiviral vectors was conducted as we have described (Xing et al., 2012). RHRSPs were anesthetized as above and positioned on a stereotactic apparatus. SiRNA preparations were injected into the right thalamus using a 15-μl Hamilton syringe at a rate of 0.1 μl/min at four separate sites (3 μl per site, 12 μl per animal) following the coordinates: anterior-posterior (AP) - 2.6 mm, medial-lateral (ML) 2.6 mm, dorsal-ventral (DV) −6.4/-6.6 mm, and AP - 3.0 mm, ML 2.8 mm, DV −6.2/-6.4 mm in relative to bregma. To keep the virus from leaking, the needle was retained for 15 min before withdrawn. The animals received MCAO surgery at 14 days post-injection to enable sufficient gene expression.

### Bromodeoxyuridine labeling

To labeling the dividing endothelial cells, all animals received intraperitoneal injection of bromodeoxyuridine (BrdU) (B9285, Sigma-Aldrich), dissolved in 0.9 % sodium chloride, at a dose of 50 mg/kg twice daily starting 24 h after MCAO for 6 consecutive days as previously reported (Xing et al., 2019).

### Morris water maze test

The Morris water maze task was conducted to assess cognitive function for six animals randomly selected from the respective groups as previous reported (Xiao et al., 2022). On the second day after MCAO, animals underwent 60 s of adaptive training to acclimate to the pool. Spatial acquisition training was performed on day 3–6 after MCAO, allowing animals to start from a position to climb onto the platform within 60 s with a 10-cm diameter platform under the water. Animals were trained four times each day with a 15-minute interval between sessions. The time for seeking the platform referred as to escape latency and swimming distance were recorded. On day 7, the platform was taken away and the animals were free to swim for 60 s. The time spent in the target quadrant and numbers of crossing the original location of the platform were recorded using a video tracking device to assess the degree of spatial memory.

### Brain tissue preparation

At 7 days after MCAO, six animals from the respective group were randomly selected to be sacrificed under deep anesthesia, and then transcardially perfused with 0.9 % sodium chloride followed by 4 % paraformaldehyde (PFA) in 0.1 M PBS (pH 7.4). Subsequently, the brains were removed and kept in PFA solution for 12 h, and sequentially dehydrated in 20 % and 30 % sucrose solution overnight at 4°C. Coronal sections with 10-μm thickness from + 4.7 to −5.2 mm in relative to bregma were made on a cryostat (CM1900, Leica).

### Nissl staining

A series of brain sections (bregma +4.7 ∼ −5.2 mm) were selected from the respective group for Nissl staining to determine primary infarct volumes as previously described (Xing et al., 2019). Sections were stained using 0.3 % cresyl violet (C0117, Beyotime) according to the standard protocol. Images were then captured with a section scanner (KF-PRO-020, Kfbio). Primary infarct volume was quantified as a percentage of the respective contralateral hemisphere volume.

### Immunofluorescent staining

Another series of sections from the individual group were chosen to perform immunofluorescent analysis. Sections were blocked with blocking buffer (P0260, Beyotime) for one hour at room temperature, and then incubated with the primary antibodies overnight at 4°C as follows: mouse anti-Beclin1 (66665–1, Proteintech Group), mouse anti-BrdU (B2531, Sigma-Aldrich), rabbit anti-CD31 (ab281583, Abcam), rabbit anti-LC3 (PM036, MBL), mouse anti-LC3 (E5Q2K, Cell Signaling Technology), rabbit anti-Laminin (L9393, Sigma-Aldrich), mouse anti-NeuN (24307S, Cell Signaling Technology), mouse anti-GFAP (ab4674, Abcam), mouse anti-angiopoietin-1 (ANG-1; sc517593, Santa Cruz) and rabbit anti-angiopoietin-2 (ANG-2; sc74403, Santa Cruz). For BrdU staining, additional pretreatment was performed with 2 N HCl for 30 min and 0.1 M boric acid solution in PBS. The sections were then incubated with goat anti-mouse IgG (ab150077; Abcam) and goat anti-rabbit IgG (ab150080; Abcam) at room temperature for one hour. Fluorescent signals were detected using a fluorescence microscope (Olympus, BX51).

### Isolation of thalamic vessels

To specifically investigate vascular autophagy, animals were randomly selected from each group for isolating blood vessels from the thalamus as we have previously described (Xiao et al., 2022). Briefly, animals were treated with transcardial perfusion using cold 0.9 % sodium chloride, and the ipsilateral thalamus was rapidly dissected and homogenized in 2 ml of cold MCDB131 medium (10372019, Thermo Fisher Scientific) with an organizational processor (Miltenyi). After centrifugation at 2000 × g for 5 min at 4°C, the supernatant was removed and the pellet was resuspended in 1 ml of 15 % (wt/vol) cold dextran-Dulbecco’s phosphate-buffered saline (DPBS) (dextran: 31390, Sigma Aldrich; DPBS: 14190250, Thermo Fisher Scientific). The pellet was gently sluiced and centrifuged at 10,000 × g for 15 min at 4°C. After removing the supernatant, the pellet was resuspended in dextran-DPBS as above. These steps were repeated three times to obtain the vessel-containing pellets for immunoblotting analysis.

### Immunoblotting analysis

Protein samples were extracted from thalamus vessels using ice RIPA lysis buffer (9806, Cell Signaling Technology), containing protease and phosphatase inhibitors (539132–1SETCN, Millipore). Protein concentration was determined using the BCA protein assay system (Pierce). Twenty micrograms of proteins were separated by SDS-PAGE and then transferred to PVDF membrane (ISEQ00010, Millipore). The membranes were blocked with 5 % nonfat milk and incubated with primary antibodies overnight at 4°C as follows: mouse anti-Beclin1 (66665–1, Proteintech Group), rabbit anti-LC3 (PM036, MBL), rabbit anti-ANG-1(23302–1-AP, Proteintech Group), rabbit anti-ANG-2 (ab155106, Abcam), rabbit anti-VEGF (WL00009b, Wanleibio) and rabbit anti-GAPDH (2118S, Cell Signaling Technology). After washing, the membranes were incubated with the respective secondary antibodies: goat anti-rabbit IgG (7074, Cell Signaling Technology) and goat anti-mouse IgG (7076S, Cell Signaling Technology). Protein bands were detected using enhanced chemiluminescence (P10300, NCM Biotech) and visualized by a chemiluminescence system (WBKLS0100, Millipore).

### Image data processing

For immune-positive cell counting, three non-overlapping fields of view (400 x magnification) were selected within the thalamus. The ratio of Laminin-labeled fluorescence area relative to total area was used as an index for blood vessel density. For immunoblotting analysis, the ratios of band gray value for target protein relative to GAPDH were determined. All quantitative analyses were implemented with Image J software (Bethesda).

### Statistical analysis

All data were analyzed using GraphPad Prism (version 9.0). Normal distributed data was presented as mean ± standard deviation and analyzed by Student's *t*-test. Non-normally distributed data was presented as median ± interquartile range and analyzed by the Mann-Whitney *U* test. Multiple comparisons were analyzed by one-way ANOVA. Statistical significance was set at *P* < 0.05.

## Results

### Secondary thalamic damage and behavioral performance

**Fig. 1:**
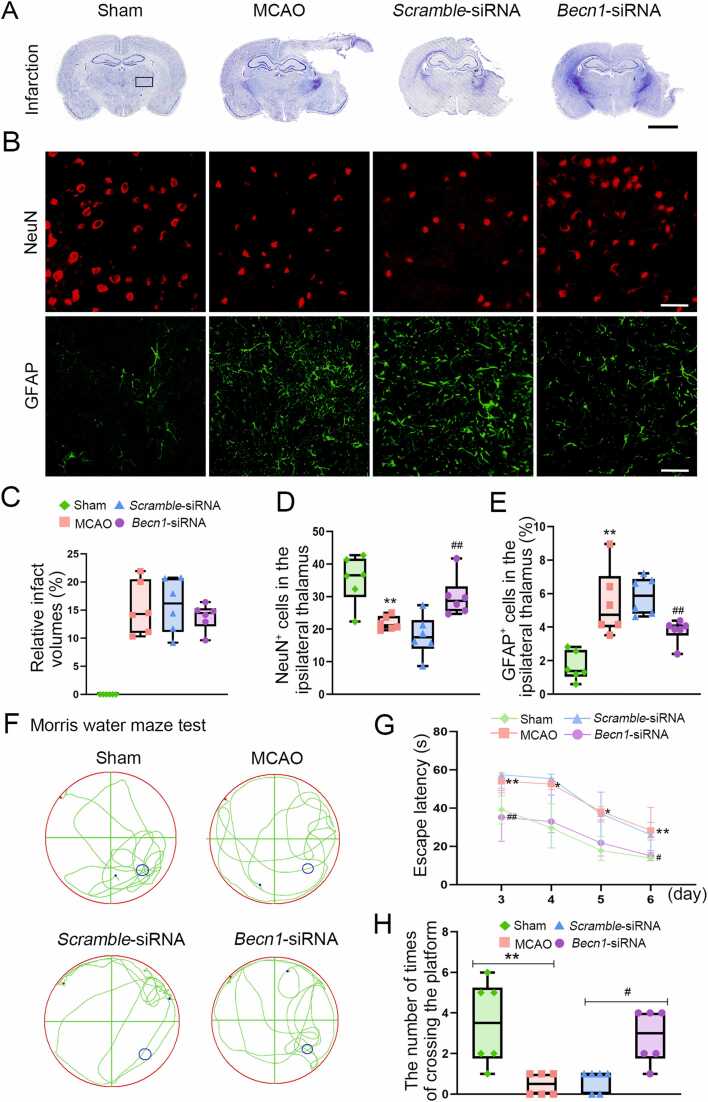
Secondary thalamic neuronal damage and behavioral performance. (A) Representative Nissl staining results showing the location of cerebral infarction in different groups. Black rectangle indicating the ipsilateral thalamus sampled for immunofluorescent pictures. Scale bar: 5 mm. (B) Immunostaining of NeuN^+^ neurons and GFAP^+^ astrocytes within the ipsilateral thalamus. Scale bar: 50 μm. (C) Quantitative analysis of infarct volumes across the MCAO, *Scramble*-siRNA and *Becn1*-siRNA groups. (D and E) Quantitative analysis of NeuN^+^ and GFAP^+^ cells. *n* = 6, data are expressed as median ± interquartile range. ^**^P < 0.01, compared with the sham-operated group; ^*##*^*P* < 0.01, compared with the *Scramble*-siRNA group. (F) Representative swimming trajectories of animals at 7 days after MCAO in different groups. (G) Escape latency for animals in different groups on day 3–6 after MCAO. *P < 0.05, ^**^P < 0.01*,* compared with the sham-operated group; ^*#*^*P* < 0.05, ^*##*^*P* < 0.01, compared with the *Scramble*-siRNA group. (H) The number of platform crossing in different groups on day 7 after MCAO. *n* = 6, data are expressed as median ± interquartile range. ^**^P < 0.01, compared with the sham-operated group; ^*#*^*P* < 0.05, compared with the *Scramble*-siRNA group.

At 7 days after MCAO, primary infarction was observed to restrict in the right cerebral cortex with the ipsilateral thalamus intact, where is sampled for all immunofluorescent analyses (Fig. 1A). The relative infarct volumes were 15.3 ± 4.7 %, 15.8 ± 4.7 % and 13.8 ± 2.3 % in the MCAO, Scramble-siRNA and *Becn1*-siRNA groups, respectively. No significant difference was found among the three groups (all *P* > 0.05; Figure1C). In the ipsilateral thalamus, there was an obvious reduction of NeuN^+^ neurons and a considerable increase in GFAP^+^ astrocytes at 7 days after MCAO in relative to the sham-operated group (both *P* < 0.01, Figure1B, D and E), indicating the secondary neuronal injury of the ipsilateral thalamus. In contrast, *Becn1*-siRNA treatment led to a significant increase in the number of NeuN^+^ neurons (*P* < 0.01; Figure1B and D) and a marked decrease in GFAP^+^ astrocytes in the ipsilateral thalamus at 7 days after MCAO (*P* < 0.01; Figure1B and E).

The results of Morris water maze test showed that animals in the MCAO group had longer latency to find the escape platform compared to the sham-operated group from day 3–6 after MCAO (all *P* < 0.05; Fig. 1F and G). Additionally, animals in the MCAO group had decreased numbers of platform crossing when compared to the sham-operated group on the seventh day after MCAO. (*P* < 0.01, Fig. 1F and H). In contrast, animals treated with the *Becn1*-siRNA group showed shorter escape latencies and a greater number of platform crossing than those in the Scramble-siRNA group at 7 days after MCAO (both *P* < 0.05; Fig. 1F-H).

### Expression of Beclin1 and autophagy in the ipsilateral thalamic vessels following cerebral infarction

**Fig. 2:**
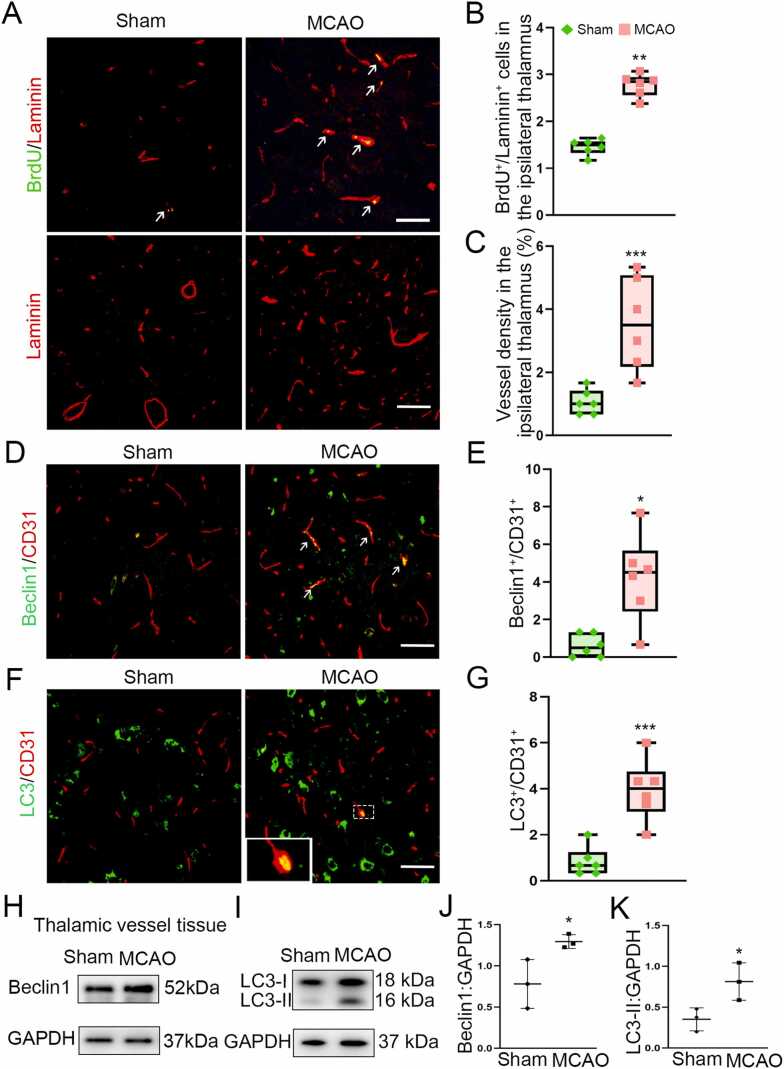
Expression of Beclin1 and autophagy in the ipsilateral thalamic vessels after cerebral infarction. (A) Immunostaining of BrdU-positive cells and Laminin-labeled blood vessels in the ipsilateral thalamus at 7 days after MCAO. Scale bar: 50 μm, 100 μm. (B and C) Quantitative analysis of BrdU^+^/Laminin^+^ cells and density of blood vessels. *n* = 6, data are expressed as median ± interquartile range. ^**^P < 0.01, ^***^P < 0.001, compared with the sham-operated group. (D) Double-staining of Beclin1 and CD31 in the ipsilateral thalamus (arrows). Scale bar: 50 μm. (E) Quantitative analysis of Beclin1^+^/CD31^+^ cells. *n* = 6, data are expressed as median ± interquartile range. *P < 0.05, compared with the sham-operated group. (F) Co-staining of LC3 with CD31 in the ipsilateral thalamus. Scale bar: 50 μm. (G) Quantitative analysis of LC3^+^/CD31^+^ cells. *n* = 6, data are expressed as median ± interquartile range. ^***^P < 0.001, compared with the sham-operated group. (H and I) Immunoblotting shows Beclin1 and LC3-II expression in the isolated thalamic vessels. (J and K) Quantitative analysis of Beclin1 and LC3-II levels relative to GAPDH. *n* = 3, data are expressed as mean ± standard deviation. *P < 0.05, compared with the sham-operated group.

Immunofluencent results showed that BrdU/Laminin-positive cells were obviously observed in the ipsilateral thalamus in relative to the sham-operated group at 7 days after MCAO (Fig. 2A). The number of BrdU^+^/Laminin^+^ cells was significantly increased in the MCAO group as compared with that in the sham-operated group (*P* < 0.01, Fig. 2B). The density of Laminin-labeled blood vessels in the MCAO group was markedly higher than that in the sham-operated group (*P* < 0.001, Fig. 2A and C). We conducted immunofluorescent staining of CD31 and Laminin to examine the expression of Beclin1 or LC3 on endothelial cells and the changes of vascular density. In parallel, Beclin1 was shown to be colocalized with CD31^+^ endothelial cells in the ipsilateral thalamus at 7 days after MCAO, and the number of Beclin1^+^/CD31^+^ cells was markedly increased compared to the sham-operated group (*P* < 0.05, Fig. 2D and E). Additionally, the number of LC3^+^/CD31^+^ cells in the MCAO group was significantly increased compared with that in the sham-operated group (*P* < 0.001, Fig. 2F and G). The results of immunoblotting analysis demonstrated that the levels of Beclin1 and LC3-II in the isolated vessel tissues of the ipsilateral thalamus were significantly elevated at 7 days after MCAO compared to the sham-operated group (both *P* < 0.05, Fig. 2H-K). Collectively, these results suggest a possible link between angiogenesis and Beclin1-related autophagy in the thalamus after cerebral infarction.

### Beclin1 knockdown suppressed vascular autophagy and promoted angiogenesis in the ipsilateral thalamus after cerebral infarction

**Fig. 3:**
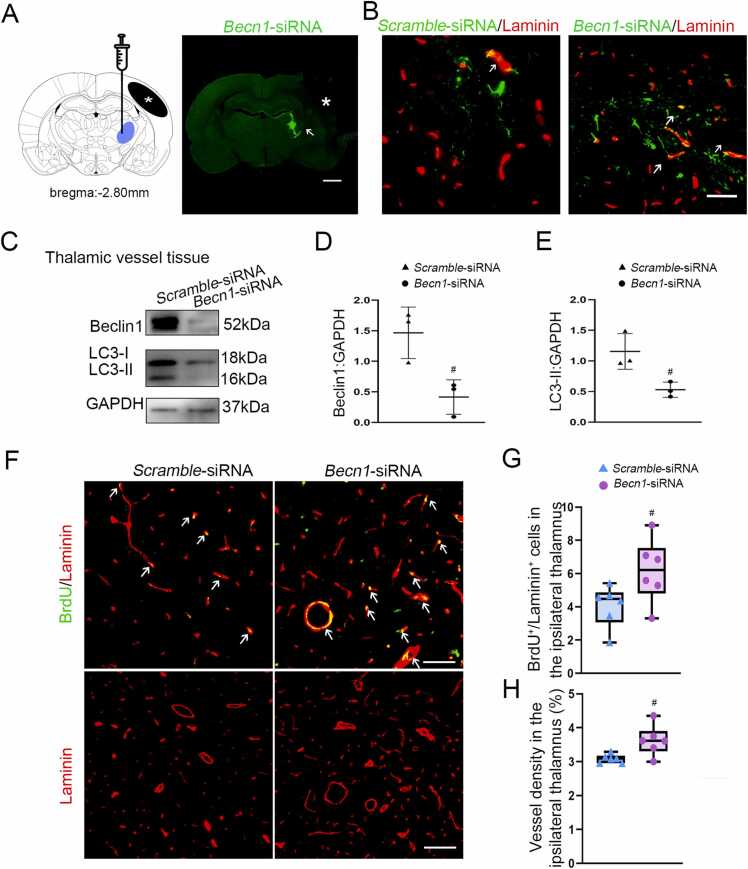
Beclin1 knockdown inhibited vascular autophagy and enhanced angiogenesis in the ipsilateral thalamus after cerebral infarction. (A) Schematic diagram of brain section (bregma: −2.8 mm) showing cortical infarction (black region) and the ipsilateral thalamus (blue region). Representative immunofluorescent slice demonstrating GFP-tagged viral vectors in the ipsilateral thalamus (arrow) at 7 days after MCAO (white star indicating the location of infarct). Scale bar: 2 mm. (B) Double-immunofluorescence revealing GFP-tagged *Scramble*- and *Becn1*-siRNA to be colocalized with Laminin-labeled blood vessels at 7 days after MCAO. Scale bar: 50 μm. (C) Immunoblotting showing the expression of Beclin1 and LC3-II in the isolated thalamic vessel tissues. (D and E) Quantitative analysis of Beclin1 and LC3-II expression relative to GAPDH. *n* = 3, data are expressed as mean ± standard deviation. ^#^*P* < 0.05, compared with the *Scramble*-siRNA group. (F) Immunostaining for BrdU and Laminin in the ipsilateral thalamus of the *Scramble*- and *Becn1*-siRNA groups. Scale bar: 50 μm, 100 μm. (G) Quantitative analysis of BrdU^+^/Laminin^+^ cells and density of blood vessels. *n* = 6, data are expressed as median ± interquartile range. ^#^*P* < 0.05, compared with the *Scramble*-siRNA group.

Next, we explored the causal relationship between Beclin1 and autophagic activation and angiogenesis by using lentivirus-mediated knockdown of Beclin1 expression. SiRNA vectors were delivered into the right thalamus two weeks before MCAO operation, and the GFP-tagged viral vectors were predominantly detected within the ipsilateral thalamus at 7 days after MCAO (Fig. 3A). Double-immunofluorescence analysis showed that GFP-tagged viral vectors were significantly colocalized with Laminin-labeled blood vessels in the ipsilateral thalamus at 7 days after MCAO (Fig. 3B). Immunoblotting showed that the levels of Beclin1 were markedly reduced in the vascular isolation of the ipsilateral thalamus in the *Becn1*-siRNA group relative to the Scramble-siRNA group at 7 days after MCAO (*P* < 0.05, Fig. 3C and D). In parallel, the conversions of LC3-I to LC3-II were synchronously decreased in the thalamic vascular isolation of the *Becn1*-siRNA group as compared with those in the Scramble-siRNA group (*P* < 0.05, Fig. 3C and E). Additionally, the number of BrdU^+^/Laminin^+^ cells was significantly increased in the ipsilateral thalamus of the *Becn1*-siRNA group compared to the Scramble-siRNA group at 7 days after MCAO (*P* < 0.05, Fig. 3F-H). Furthermore, *Becn1*-siRNA treatment led to a marked increase in the density of Laminin-labeled blood vessels in the ipsilateral thalamus in relative to the Scramble-siRNA group (*P* < 0.05, Fig. 3F and G). Altogether, these data suggest that inhibition of autophagic activation in blood vessels by Beclin1 knockdown enhances angiogenesis in the ipsilateral thalamus after cortical cerebral infarction.

### Beclin1 knockdown and alterations of angiopoietin-1/2 and VEGF in the ipsilateral thalamus after cerebral infarction

**Fig. 4:**
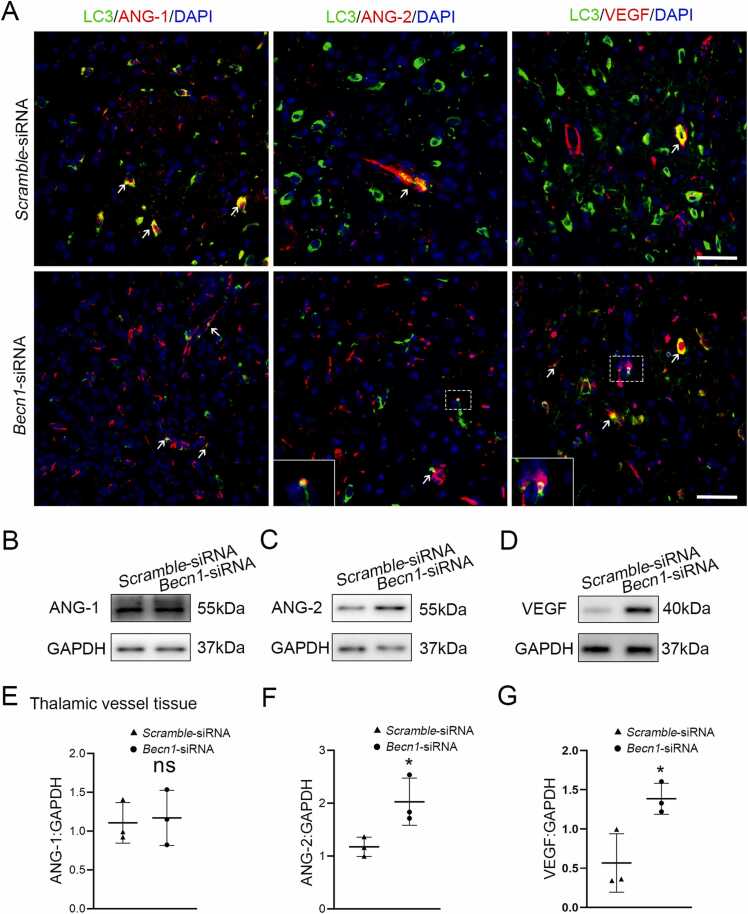
Beclin1 knockdown and alterations of angiopoietin-1/2 and VEGF in the ipsilateral thalamus after cerebral infarction. (A) Immunofluorescence results showing co-localization of LC3 with ANG-1, ANG-2 and VEGF in the ipsilateral thalamus. Scale bar: 50 μm. (B-D) Immunoblotting demonstrating the levels of ANG-1, ANG-2, and VEGF in the thalamic isolations. (E-G) Quantitative analysis of ANG-1, ANG-2 and VEGF levels relative to GAPDH. *n* = 3, data are expressed as mean ± standard deviation. *P < 0.05, compared with the *Scramble*-siRNA group.

To explore the potential mechanisms of Beclin1-mediated autophagy in angiogenesis, we further examined the possible alterations of angiogenesis-related factors. Immunofluorescent results showed that LC3 was co-localized with ANG-1, ANG-2 and VEGF in the ipsilateral thalamus of the *Becn1*- and Scramble-siRNA groups (Fig. 4A). Additionally, immunoblotting results revealed no significant difference in the level of ANG-1 within the thalamic isolations of the *Becn1*-siRNA group compared to the Scramble-siRNA group (*P* > 0.05, Fig. 4B and E). In contrast, the levels of ANG-2 and VEGF were markedly elevated in the vascular isolations of the *Becn1*-siRNA group compared to the Scramble-siRNA group (both *P* < 0.05, Fig. 4C, F and G). Collectively, these results implicate that inhibition of vascular autophagy restores the levels of ANG-2 and VEGF rather than ANG-1.

## Discussion

This study demonstrated autophagic activations in endothelial cells along with enhanced angiogenesis in the ipsilateral thalamus after cerebral infarction. Inhibition of autophagic activation in endothelial cells by downregulation of Beclin1 promoted the degree of angiogenesis and rescued the secondary neuronal loss and gliosis in the ipsilateral thalamus. This effect was accompanied by an improvement of cognitive decline. Additionally, suppression of autophagic activity in blood vessels of the ipsilateral thalamus by Beclin1 knockdown markedly increased the levels of VEGF and ANG-2 after cerebral infarction. Taken together, these findings suggest that Beclin1-mediated vascular autophagy negatively relates to angiogenesis in the thalamus, possibly through regulating the levels of ANG-2 and VEGF, thereby contributing to the secondary neuronal damage of the thalamus and cognitive improvement following cerebral infarction.

Focal cerebral infarction leads to the secondary thalamic damage, which develops chronically and progressively after stroke and impedes post-stroke neurological function recovery (Cao et al., 2020). In this study, we chose the RHRSP model to mimic human stroke etiology while avoiding the confounding effects associated with genetic defects as seen in the stroke-prone spontaneously hypertensive model Zeng et al., 1998). Besides, focal cortical infarct in the MCAO model was restricted in the neocortex with the ipsilateral thalamus intact, allowing for mechanistic explorations of the secondary damage.

Autophagy degrades and recycles cytoplasmic substances via lysosomes to maintain cellular and tissue homeostasis. Endogenous autophagy in endothelial cells regulates cellular responses to diverse stressors, which is crucial for maintaining redox homeostasis and endothelial cell plasticity (Schaaf et al., 2019). The relationship between autophagy and angiogenesis may vary with different pathologic situations. A line of evidence suggests cellular autophagy to be involved in angiogenesis in diabetes models (Xie et al., 2011, Du et al., 2017). In contrast, other study showed that autophagic defects could inhibit lymphangiogenesis (Meçe et al., 2022). Previously, we have demonstrated that inhibition of autophagic flux in blood vessels correlated with the degree of angiogenesis in the thalamus by RTN4 knockdown after cerebral infarction (Xiao et al., 2022). Consistently, the present study further confirmed the causal effect of autophagic activation in blood vessels on angiogenesis by downregulation of Beclin1, which exerts an essential role in autophagic induction by assembling class III phosphatidylinositol 3-kinase complexes (Tran et al., 2021). We found a synchronized elevation of Beclin1 and LC3-II expression on endothelial cells within the ipsilateral thalamus after cerebral infarction, suggesting the plausible relationship between Beclin1 and vascular autophagy in the thalamus. Further, knockdown of Beclin1 markedly inhibited autophagic activation in blood vessels and increased the number of BrdU/Laminin-positive cells as well as the degree of vessel density in the ipsilateral thalamus. Given that BrdU labeling is reflective of cell prefoliation (Wojtowicz and Kee, 2006), these results suggest that Beclin1-mediated autophagic activation in blood vessels negatively regulates angiogenesis occurring in the thalamus after cerebral infarction.

The mechanisms underlying the involvement of autophagy in angiogenesis remain unclear. Angiogenesis is principally regulated by pro- and anti-angiogenic factors (Zhu et al., 2010, Sedighi et al., 2023). In a mouse melanoma tumor model, it has been shown that increased angiogenesis in Beclin1-deficient mice primarily related to an elevation of erythropoietin rather than VEGF (Lee et al., 2011). Inconsistently, we found that VEGF was colocalized with the autophagy marker LC3, and knockdown of Beclin1 resulted in an increase of VEGF level in the thalamic vessels, suggesting a possible link of autophagy with VEGF expression. This is supported by the previous study showing that blockade of autophagic pathway rescued cell death in VEGF-knockdown human umbilical vein endothelial cells (Lee et al., 2007, Domigan et al., 2015). Additionally, the current results showed that ANG-1 and ANG-2 were colocalized with LC3-positive cells in the thalamus, and knockdown of Beclin1 markedly increased the level of ANG-2 rather than ANG-1 in blood vessels within the ipsilateral thalamus. These results imply the potential correlate of vascular autophagy with ANG-2 in addition to VEGF. ANG-2 along with ANG-1 are the important members of angiopoietins (Akwii et al., 2019). Functionally, ANG-1 is suggested for the maintenance of vascular quiescence and vascular maturation by inducing TIE-2 to accumulate at cell-cell junctions for maintaining endothelial cell quiescence (Fagiani and Christofori, 2013). In contrast, ANG-2 functions as a competitive antagonist to ANG-1 to facilitate vascular sprouting (Fagiani and Christofori, 2013). Along with these previous studies, our findings suggest that inhibition of autophagy might reduce the degradation of VEGF and ANG-2, thereby coordinately promoting angiogenesis in the thalamus after cerebral infarction. Nevertheless, the effects of autophagy on the regulation of angiogenic factors are required to be further elucidated using Beclin1 knockout mice.

In the present study, knockdown of Beclin1 was found to correlate with an improvement of cognitive impairment after cerebral infarction. This effect might be explained from two aspects. It has been showed that promoting angiogenesis could directly facilitate neurological function recovery after stroke (Nih et al., 2018, Wang et al., 2019, Janyou et al., 2020). We found that inhibition of autophagy by Beclin1 knockdown markedly enhanced angiogenesis, which might directly contributes to improvement of cognitive function as previously reported (Xiao et al., 2022). Alternatively, our data demonstrated that knockdown of Beclin1 significantly rescued the degree of neuronal loss and astrogliosis in the thalamus. Given that the thalamus closely links to cognitive function (Shine et al., 2023), we postulate that the cognitive improvement might indirectly related to the attenuations of neuronal damage of the thalamus as resultant of angiogenesis induced by Beclin1 knockdown.

In conclusion, the current findings indicate that Belin1-mediated autophagic activation in blood vessels negatively regulates angiogenesis and the secondary neuronal damage in the thalamus, thereby contributing to cognitive impairments after cerebral infarction. The neuroprotective effect might relate to restoration of ANG-2 and VEGF mediated by inhibiting vascular autophagy in the thalamus.

## CRediT authorship contribution statement

**Mengzhi Liu:** Writing – original draft, Validation, Investigation, Formal analysis, Data curation, Conceptualization. **Yuqian Chen:** Methodology, Investigation, Formal analysis, Conceptualization. **Xinyan Fan:** Methodology, Investigation. **Jinmin Gu:** Resources, Methodology. **Shihui Xing:** Writing – review & editing, Supervision, Project administration, Funding acquisition.

## Declaration of Competing Interest

The authors declare that they have no known competing financial interests or personal relationships that could have appeared to influence the work reported in this paper.
